# Plantar Pressure Responses to Backpack Load in Long-Distance Hikers: A Cross-Sectional Observational Study

**DOI:** 10.3390/jfmk11010036

**Published:** 2026-01-15

**Authors:** Coral Moya-Cuenca, Sara Zúnica-García, Alba Gracia-Sánchez, Santi García-Cremades, Ana María Oltra-Romero, Esther Chicharro-Luna

**Affiliations:** 1 Department of Behavioral Sciences and Health, Nursing Area, Faculty of Medicine, Miguel Hernández University of Elche (UMH), 03550 Alicante, Spain; cmoya@umh.es (C.M.-C.); szunica@umh.es (S.Z.-G.); ana.oltra@umh.es (A.M.O.-R.); ec.luna@umh.es (E.C.-L.); 2 Centre for Operations Research, Miguel Hernández University of Elche (UMH), 03202 Elche, Spain; jose.garciac@umh.es; 3 Institute of Health and Biomedical Research of Alicante (ISABIAL), 03010 Alicante, Spain

**Keywords:** backpack load, plantar pressure, biomechanics, long-distance hikers, trekking

## Abstract

**Background:** Long-distance hiking usually requires carrying a backpack, adding external load to the lower limbs and modifying plantar loading patterns. Excessive loads may contribute to overuse injuries, but quantitative evidence to support current recommendations on backpack weight is still scarce. This study aimed to examine how different backpack loads influence plantar pressure in long-distance hikers. **Methods:** A cross-sectional observational study was conducted in adults who had walked at least 20 km during the previous 24 h. Sociodemographic and clinical variables were recorded, and barefoot plantar pressure was assessed using the Podoprint^®^ system under four conditions: without a backpack, with the habitual backpack, and with backpacks loaded to 10% and 20% of body weight. Static and dynamic plantar pressure parameters were analyzed using repeated-measures comparisons. **Results:** A progressive increase in plantar force was observed in both feet as backpack load increased. Compared with the unloaded condition, static forefoot pressure rose by 5.41% with a 10% load and by 8.73% with a 20% load (*p* = 0.005); rearfoot pressure increased by 5.01% and 10.17% (*p* = 0.015); and total foot pressure by 5.04% and 9.61% (*p* = 0.002). Loads above 10% of body weight significantly modified static plantar pressures and were associated with measurable changes during dynamic assessment. **Conclusions:** In long-distance hikers, carrying a backpack that exceeds approximately 10% of body weight leads to a clear, load-dependent increase in plantar pressure. These findings provide biomechanical support for recommendations that advise limiting backpack load to around 10% of body weight to reduce plantar stress during hiking.

## 1. Introduction

Hiking is a widely practiced recreational activity worldwide, with increasing participation in outdoor leisure and nature-based tourism in environments such as national parks and wildlife reserves [1,2]. In addition to helping individuals meet physical activity recommendations, hiking offers the added benefit of exposure to natural settings. However, the growth of these activities has been accompanied by a rise in musculoskeletal injuries related to outdoor pursuits over recent decades [3,4].

In most Western countries, hikers typically carry their equipment in a backpack [5]. Prolonged exposure to excessive external load can alter individual biomechanics and contribute to postural alterations [6,7]. Several studies have identified a direct relationship between backpack carriage and the plantar pressure exerted on the feet [8,9,10,11,12]. Heavier backpack loads have also been associated with a higher likelihood of foot blisters, one of the most frequent problems reported by hikers [13,14].

Carrying heavy loads has further been linked to an increased risk of musculoskeletal disorders [5]. As external load rises, joint loading also increases [5,15], and greater load-bearing is associated with longer stance time and shorter stride length, both of which may impair normal foot function [16]. Although it has been suggested that backpack weight should not exceed 10–15% of body weight, recommendations commonly cited in the literature suggest keeping carried loads within this range; however, the specific mechanical effects on the feet are not yet fully understood [15,17].

The foot is composed of multiple segments that function as an integrated multi-segment structure, providing stability, shock absorption, and propulsion during gait [18]. Changes in loading conditions can modify posture and foot function and can be objectively quantified using baropodometric analysis [19]. Pressure platforms make it possible to measure the magnitude and distribution of forces acting on the plantar surface under both static and dynamic conditions [12,20,21]. Furthermore, large normative datasets reporting spatiotemporal and plantar pressure patterns across the lifespan support age-aware interpretation of pedobarographic outcomes [22].

Research conducted in occupational and tactical settings has shown that heavier equipment leads to measurable increases in plantar pressure and contact area in static and dynamic evaluations, reflecting flattening of the longitudinal and transverse arches and greater mechanical strain on the foot [23]. These alterations appear to be more marked in individuals with flat-foot morphology, who demonstrate earlier and greater arch collapse under load [23]. In addition to total load, load distribution itself significantly influences plantar pressure patterns and postural control, highlighting the relevance of ergonomic and symmetrical load carriage [23].

Experimental studies have further demonstrated that backpack load induces quantifiable changes in plantar loading. In adults walking with loads ranging from 0 to 30% of body weight, the first vertical ground reaction force peak increased proportionally with both load magnitude and gait speed [24]. Other investigations have also reported load-related increases in regional peak plantar pressures (e.g., hallux/metatarsal heads/heel) during walking under backpack loads [25]. Similarly, research in younger participants found that backpacks representing 10–20% of body weight produced significant increases in midfoot contact area and relative impulse in the metatarsal regions during gait [26]. However, much of the available pedobarographic evidence derives from school-aged or tactical/occupational cohorts, and the transferability of these findings to recreational adults remains uncertain.

Despite these contributions, the existing evidence presents important limitations. Most studies have been conducted either in children or in highly trained populations such as military personnel, law enforcement officers, or firefighters, which may limit generalizability to recreational adults. Children show immature musculoskeletal development and different gait patterns compared with adults; therefore, their plantar pressure responses to load do not necessarily reflect those of mature feet. In contrast, tactical populations undergo selection and conditioning and are repeatedly exposed to heavy loads, leading to adaptations in posture and load-carrying efficiency that are not representative of recreational hikers. Moreover, operational demands (e.g., speed marching, uneven terrain, prolonged exposure, performance constraints) differ markedly from leisure hiking, where participants vary widely in age, fitness, backpack fit, and experience. For these reasons, mechanical responses to load cannot be assumed to apply directly to the broader adult recreational population. In addition, plantar pressure and spatiotemporal gait parameters vary across the lifespan, further supporting the need for age-aware interpretation when studying heterogeneous recreational cohorts [22].

Therefore, the aim of this exploratory study was to quantify within-subject changes in static and dynamic plantar pressure outcomes across graded backpack-load conditions in recreational hikers under ecologically relevant walking conditions. We hypothesized a progressive, load-dependent increase in plantar pressures, with a potential transition in response around commonly cited load ranges (≈10–15% of body weight). To capture complementary information from standing and gait, we included both static assessment and dynamic multi-step measurements, using standardized load conditions (including 10% and 20% body weight) alongside participants’ habitual loads.

## 2. Materials and Methods

### 2.1. Study Design and Ethical Approval

This observational cross-sectional study was conducted in accordance with the Strengthening the Reporting of Observational Studies in Epidemiology (STROBE) guidelines for observational research [27,28]. The protocol was approved by the Responsible Ethics Investigation Office of Miguel Hernández University (code DCC.AGS.01.22) and the study was carried out in line with the principles of the Declaration of Helsinki [29,30]. This study did not involve any health-related intervention; therefore, clinical trial registration was not applicable.

### 2.2. Participants and Recruitment

Participants were recruited in July 2022 at a pilgrims’ hostel on the Camino de Santiago, a common stop for long-distance hikers. During their stay at the hostel, hikers were invited to participate and received verbal and written information about the aims and procedures of the study. Written informed consent was obtained from all participants before any data were collected, ensuring voluntary participation and confidentiality.

Non-random consecutive sampling was used. Inclusion criteria were: (a) age ≥ 18 years; (b) having walked at least 20 km during the previous 24 h; and (c) having carried a backpack during that stage. Participants were excluded if they had undergone lower-limb or major musculoskeletal surgery in the previous six months.

The final sample consisted of 39 hikers (21 men, 18 women), with a mean age of 36.57 ± 13.96 years. Men were generally taller and heavier than women, resulting in higher BMI values (Table 1). Participants represented multiple nationalities: 43.6% Spanish, 23.1% Italian, 10.3% American, 5.1% Brazilian, 5.1% Mexican, and 13.0% from other countries. Most participants (87.2%) reported regular physical activity, although only 53.8% had specifically trained for a hiking route. Data collection was performed by two investigators with clinical experience in biomechanics and podiatric assessment.

Participants were classified as recreational hikers based on the following operational criteria:

(a) engagement in hiking activities for leisure rather than competitive or occupational purposes;
(b) absence of prior professional mountain training;
(c) typical hiking frequency of 1–4 outings per month;
(d) no systematic load-carriage conditioning.

A detailed characterization of experience level was considered necessary because biomechanical adaptation to load carriage depends on prior exposure, and differences in this variable may influence the plantar responses observed. Participants self-reported a median hiking experience of 3 years (range: 1–12 years). Typical distances covered during their hikes ranged from 10 to 25 km, and habitual backpack loads varied between 5% and 12% of body weight. Although 53.8% stated they had trained specifically for the Camino de Santiago, this training mainly consisted of moderate walking without structured load-carrying programs. This information was collected to contextualize biomechanical responses, as load-carrying experience is known to influence gait adaptations and plantar pressure patterns.

Sample size was calculated using Epidat 4.0 (Galicia Health Department, A Coruña, Spain). Assuming a medium effect size (Cohen’s d = 0.5), an α error of 0.05 and a statistical power of 80%, the required sample size for detecting differences in mean plantar pressure between loading conditions was estimated at 34 participants. To compensate for potential data loss, a total of 39 hikers were ultimately included.

### 2.3. Recent Hiking Activity and Fatigue Status

All participants had walked at least 20 km during the 24 h preceding data collection, with distances ranging between 20 and 32 km, as recorded on their pilgrim credentials or mobile tracking applications. Because plantar pressure assessment was conducted immediately upon arrival at the hostel, participants were likely experiencing residual fatigue, muscle soreness, and mechanical stress from prolonged walking. This fatigue state represents a contextual factor inherent to long-distance hiking and was therefore intentionally preserved rather than controlled experimentally.

Because fatigue can modify step length, postural control, and shock-absorption capacity, it is acknowledged that it may interact with the plantar loading responses induced by the different backpack loads. The potential influence of this residual fatigue was taken into account in both the interpretation of the results and the discussion of the study’s limitations.

### 2.4. Hiking Context and Experimental Conditions

All participants were recreational hikers who were completing the Camino de Santiago on foot as a leisure activity, rather than as professional or organized athletes. Measurements were performed indoors at the hostel, on a flat, hard surface, under stable environmental conditions to minimize variability associated with terrain irregularities.

Participants had completed the walking stage wearing their own hiking footwear, typically low-cut hiking shoes with flexible midsoles and rubber outsoles. However, plantar pressure assessments were conducted barefoot to obtain direct measurements of foot–ground interaction.

Importantly, the assessments were not performed immediately after the hiking stage. Upon arrival at the hostel, participants first completed baseline procedures, including weighing their habitual backpack to document the actual load carried during the stage. This measurement was taken before showering or resting to ensure accurate recording of the real hiking load.

After this initial registration, all participants followed their usual post-stage routine showering, eating, and resting for a variable period before taking part in the experimental plantar pressure assessments later in the afternoon. This approach ensured that the simulated load conditions (no backpack, habitual backpack, 10% load, and 20% load) were tested under safe and standardized circumstances. At the same time, the procedure still preserved the influence of residual, ecologically realistic fatigue resulting from walking 20 km or more during the previous 24 h, consistent with the physical demands of the Camino de Santiago.

Each participant was evaluated under four backpack load conditions in a fixed order: (1) no backpack; (2) habitual backpack; (3) backpack loaded to 10% of body weight; and (4) backpack loaded to 20% of body weight. The 10% and 20% loads were simulated using calibrated weight bags of varying sizes (comparable to small rice sacks), which were combined and evenly distributed inside a standard hiking backpack to reproduce realistic load placement. Shoulder and waist straps were individually adjusted to ensure symmetrical load distribution and a natural upright posture. Although the order of conditions was not randomized, 2 min rest intervals were provided between trials to reduce fatigue, and participants were allowed brief additional rests after each loaded condition if needed.

Foot dominance was not recorded, as both feet were assessed under identical measurement procedures. Each participant completed the full assessment protocol once, during the afternoon of the same day in which their hiking stage had been completed.

### 2.5. Plantar Pressure Assessment

Static and dynamic plantar pressure measurements were collected using a Podoprint^®^ S4 platform (Namrol Group, Barcelona, Spain), a baropodometric system with demonstrated reliability for assessing barefoot plantar pressure distribution. Previous research has reported moderate-to-good reliability for key plantar pressure parameters, with intraclass correlation coefficients (ICCs) ranging from 0.81 to 0.93 [31]. The platform measures 57 cm × 57 cm × 9 mm and contains 1600 resistive sensors. Signals were transmitted digitally to a computer and processed using dedicated software that provides plantar pressure, center of pressure and postural sway parameters.

**Figure 1:**
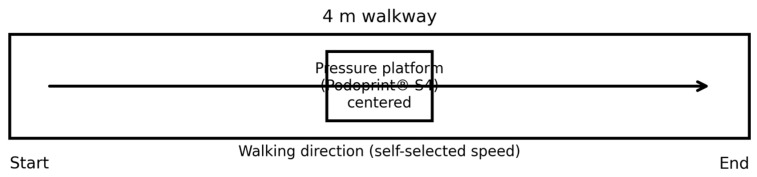
Schematic representation of the 4 m walkway with the Podoprint^®^ S4 platform positioned at the center.

All assessments were conducted indoors on a flat, hard surface, under controlled conditions. For static trials, participants stood barefoot on the platform, with feet positioned comfortably, arms relaxed alongside the body and gaze directed at a fixed point at eye level. Static recordings were collected for 30 s per condition. Static outcomes were computed over the full 30 s recording window and the final value for each condition corresponds to the mean of three recordings. Dynamic trials were performed along a 4 m walkway at a self-selected comfortable speed, with the pressure platform positioned at the center of the walkway (Figure 1). Dynamic trials were acquired using the platform’s multi-step mode. Because multi-step acquisition aims to obtain clearly captured contacts from both limbs, the duration of each dynamic recording was not fixed and depended on the participant’s walking speed and the time required to capture valid steps suitable for segmentation and analysis. Self-selected speed was chosen to reflect ecological hiking conditions and to avoid gait alterations associated with imposed pacing.

Before testing, participants removed wrist ornaments and items from their pockets and remained barefoot (no socks). Under each of the four backpack load conditions (no backpack, habitual backpack, 10% and 20% of body weight), static and dynamic measurements were obtained. For dynamic assessments, three multi-step trials were recorded per condition. Dynamic data reflect the stance phase of each captured foot contact (from initial contact to toe-off). Steps were segmented within the software and only valid contacts fully contained within the active area (without targeting) were retained. For each condition, the reported dynamic outcomes represent the mean across segmented valid steps aggregated within each trial and then averaged across the three trials.

Plantar pressure values were expressed in grams per square centimetre (g/cm^2^), which is the native output unit of the Podoprint^®^ system. For reference, 1 g/cm^2^ corresponds to 98.07 Pa (0.098 kPa) in SI units, facilitating comparison with studies reporting pressures in kilopascals. The main plantar pressure variables included peak pressure (highest pressure recorded at any sensor during stance), mean pressure (average of all activated sensors) and total contact area (cm^2^).

**Figure 2:**
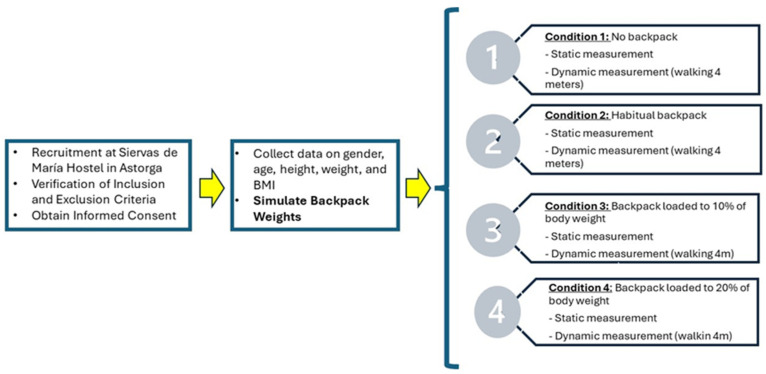
Data Collection Flow Diagram.

Both mean plantar pressure and maximum (peak) plantar pressure were analyzed. Mean pressure reflects the average pressure across activated sensors within a region, whereas peak pressure represents the highest sensor value recorded during stance and captures localized maximal loading. Plantar regions were analyzed using the Podoprint^®^ software’s (version 2.6) standard segmentation for this protocol, which provides forefoot and hindfoot regions (and whole-foot outcomes); a medial–lateral subdivision was not available for consistent extraction and export across participants and conditions. Sociodemographic and clinical variables were also collected, including sex, age, height, weight and body mass index (BMI). Height and weight were measured using an RGT^®^ stadiometer and a Seca 803 Clara digital scale (Seca GmbH & Co. KG, Hamburg, Germany). Foot posture was assessed bilaterally using the Foot Posture Index (FPI-6), which ranges from −12 (highly supinated) to +12 (highly pronated) and is reported in Table 1. Specific foot deformities or clinical diagnoses (e.g., hallux valgus, claw toes, cavus foot) were not systematically recorded and therefore were not included as covariates or stratification factors in the analyses. The overall data collection protocol is summarized in Figure 1 and Figure 2.

### 2.6. Statistical Analysis

All statistical analyses were performed using IBM SPSS Statistics (version 24.0; IBM Corp., Armonk, NY, USA). Quantitative variables are presented as mean ± standard deviation, and categorical variables as frequencies and percentages.

Normality of quantitative variables was assessed using the Kolmogorov–Smirnov test (*p* > 0.05 indicating approximate normality). Because each participant was assessed under multiple backpack-load conditions, analyses were performed within a repeated-measures (within-subject) framework, where each participant served as their own control. Pairwise comparisons were prespecified to evaluate condition-related changes relative to the unloaded condition. When assumptions for parametric testing were met, paired Student’s *t*-tests were applied; otherwise, the Wilcoxon signed-rank test for paired samples was used.

Effect sizes for pairwise comparisons were calculated using Cohen’s d for paired samples (d = t/√n) to quantify the magnitude of change independently of sample size. Values of 0.2, 0.5, and 0.8 were interpreted as small, medium, and large effects, respectively.

To provide an exploratory, sample-specific interpretive benchmark for changes in maximum plantar pressure, a one-sample Student’s *t*-test was conducted using the unloaded-condition maximum plantar pressure values as the reference. Progressive percentage increases were examined to identify the point at which differences reached statistical significance (*p* < 0.05). This procedure was used for interpretation within the present dataset and should not be considered a universal clinical threshold.

## 3. Results

Regarding backpack weight, 33.3% of participants carried a backpack weighing ≤10% of their body weight, while 38.5% carried backpacks exceeding the recommended 10% limit (Table 2).

In the static measurements, evaluation of plantar pressures (mean and maximum) on the forefoot, hindfoot, and full foot revealed consistently higher forces on the right foot compared to the left. Greater force was always exerted on the hindfoot than the forefoot.

As expected, the pressure on the forefoot and hindfoot increased with greater backpack weight. For the right foot, the average pressure increase from no backpack to 20% body weight was 0.021 kg/cm^2^ in the forefoot and 0.025 kg/cm^2^ in the hindfoot. For the left foot, the increases were 0.026 kg/cm^2^ (2549.72 Pa) and 0.029 kg/cm^2,^ respectively. Overall, the pressure on the entire foot increased by 0.023 kg/cm^2^ on the right and 0.026 kg/cm^2^ on the left. Detailed descriptive values are provided in Supplementary Table S1.

This Supplementary Table shows a progressive increase in plantar pressure and contact surface area as backpack load rises, while maintaining a stable distribution pattern between the forefoot and hindfoot. The highest pressures were consistently recorded in the hindfoot, whereas the forefoot exhibited smaller but proportional increases. The plantar contact area also expanded with additional load, increasing from approximately 135 cm^2^ to 150 cm^2^, indicating a physiological adaptation of the foot through a broader support base.

Comparing the average pressure exerted on both feet when carrying no backpack, a 10% body weight backpack, and a 20% body weight backpack, the pressure on the forefoot increased by 5.41% and 8.73% with 10% and 20% body weight backpacks, respectively (*p* = 0.005). The hindfoot showed corresponding increases of 5.01% and 10.17% (*p* = 0.015), and the full foot 5.04% and 9.61% (*p* = 0.002) (Table 3). Detailed descriptive values are provided in Supplementary Table S2.

Statistical analyses (Table 4) confirmed that mean plantar pressure increased significantly under all loaded conditions (*p* < 0.05), with effect sizes ranging from medium to large (Cohen’s d = 0.34–0.83). In contrast, maximum plantar pressure showed significant differences only for loads equal to or greater than 10% of body weight (*p* = 0.013 and *p* = 0.032), whereas the habitual backpack condition did not differ significantly from the unloaded state (*p* = 0.312).

The results of the paired Student’s *t*-tests comparing each backpack condition with the unloaded reference are presented in Table 4. Mean plantar pressure showed statistically significant increases under all loaded conditions (*p* < 0.05), with effect sizes ranging from medium to large (Cohen’s d = 0.34–0.83). In contrast, maximum plantar pressure only exhibited significant differences for loads equal to or greater than 10% of body weight (*p* = 0.013 and *p* = 0.032), while the habitual backpack condition did not differ significantly from the unloaded state (*p* = 0.312).

Regarding the magnitude of these effects, Cohen’s d values indicated small to medium effects for maximum pressure (0.16–0.42) and medium to large effects for mean pressure (0.34–0.83). These findings suggest that increasing backpack load produces progressive but moderate biomechanical changes in plantar pressure, which remain within biomechanically tolerable limits up to approximately 10% of body weight. Beyond this threshold, statistically significant differences in maximum pressure become evident, reflecting the onset of measurable alterations in plantar load distribution.

To complement the static findings, dynamic measurements were analyzed to determine the upper limit of pressure increase tolerated without significant biomechanical alteration. In the dynamic test, using the no-backpack condition as a reference, plantar pressure increased by up to 5.33% without reaching statistical significance. These values represent the highest-pressure increases tolerated before any significant biomechanical alteration is observed (Table 5). According to the pressure equation (P = F/A = m·g/A), this pressure increment would translate into an equivalent increase in body weight. When applying Earth’s gravitational constant (g = 9.8 m·s^−2^), this is equivalent to an estimated 10% increase in effective load applied to the plantar surface. This dynamic pattern is consistent with the static findings, supporting the notion that backpack loads up to approximately 10% of body weight are biomechanically tolerable.

## 4. Discussion

The results of this exploratory study suggest a progressive increase in the plantar loading (mean and peak plantar pressures) exerted on the foot as the external load increases, with the load exceeding 10% of body weight being associated with measurable changes in the maximum pressures. This pattern was observed in both static and dynamic measurements. Carrying external loads is a frequent and necessary task in hiking and occupational settings, yet its quantitative biomechanical impact on plantar pressure remains poorly defined. The present findings provide preliminary biomechanical support for a dose–response relationship between backpack load and plantar pressure, with an observed change-point around 10% of body weight in this cohort and under the present testing conditions.

As load increases, the observed rises in mean and peak plantar pressures—particularly in the hindfoot—indicate greater plantar loading demands under the present protocol. This pattern may reflect a shift toward rearfoot-dominant loading during stance. However, center-of-pressure (CoP) metrics and gait-phase–specific outcomes were not analyzed in this study, so CoP-related interpretations should be considered exploratory and addressed in future work [21].

The simultaneous increase in contact area and plantar pressure is not necessarily contradictory. Added backpack load increases the overall load applied to the foot, which can raise mean plantar pressure even if contact area also expands. Moreover, peak plantar pressure reflects localized maxima rather than an area-averaged value; thus, contact area may increase while regional peak pressures still rise due to non-uniform load redistribution.

An increase in plantar pressure has been proposed as a mechanical contributor to dermal lesions during prolonged walking, and foot blisters remain a frequent complaint in hikers [13,14]. Although the present study did not directly assess the occurrence of such lesions, the loading pattern observed here may help to contextualize the mechanical mechanisms underlying their formation. Future studies with larger samples and clinical follow-up could confirm this association.

Our results are consistent with previous studies showing a progressive increase in plantar pressure as load increases [12,25,26]. In particular, Kyung et al. (2022) [25] reported arch flattening and increased contact area when carrying equipment, which is compatible with the concomitant increases in contact area and regional plantar pressures observed here. Recent work has also reported load-related increases in regional peak plantar pressures during walking under backpack loads [25,26] further showed that loads corresponding to 10–20% of body weight can induce significant midfoot changes. Together, these findings are compatible with an early load-related transition in plantar loading responses, although the location of any ‘change-point’ is likely population- and context-dependent.

Importantly, contextual factors in this study differ from laboratory-based research and may partially explain the magnitude of the observed effects. First, participants completed a long-distance walking stage (20–32 km) within the previous 24 h. Although measurements were performed later in the afternoon after showering, eating, and resting for a variable period residual fatigue, muscle soreness, and reduced mechanical efficiency likely persisted Residual fatigue may have influenced plantar loading patterns and contributed to inter-individual variability in responses. Because CoP trajectories were not analyzed, we avoid making CoP-specific claims in the present study; nevertheless, fatigue-related modulation of plantar loading warrants dedicated investigation in future work. While this limits direct comparison with studies performed under fully rested conditions, it enhances the ecological validity of our findings by reflecting realistic hiking demands. These contextual elements help explain the magnitude and distribution of the plantar loading responses observed. Importantly, they also limit direct generalizability to fully rested conditions and to other hiking contexts.

Second, participants were recreational hikers with heterogeneous experience, and no structured load-carrying training.

Compared with tactical/professional populations that are routinely exposed to load carriage, recreational participants may have less load-specific conditioning, which could influence load-handling strategies and plantar loading responses [5,31]. In this context, the within-subject sensitivity observed near the ~10% body-weight condition may partly reflect limited exposure to structured load carriage, although other factors (e.g., prior long-distance walking and inter-individual variability in age/anthropometrics) may also contribute. This supports the need to study non-specialized adult populations separately from tactical groups, as their load exposure and compensatory strategies may differ. However, heterogeneity in age, experience, and anthropometrics may have contributed to variability in responses and limits population-level inference.

Although greater pressure was exerted in all areas, the greatest increase was recorded in the hindfoot. Similar results were obtained in the study of school-age children with flat feet, in whom a significant increase in pressure was observed in the lateral and medial areas of the heel as greater backpack weight increased [25].

The predominance of heel loading observed in our sample suggests that early-stance loading patterns play a critical role in the redistribution of plantar pressure as load increases. This mechanical pattern could explain why hindfoot pressures were more sensitive to added weight than those in the forefoot.

Both Paul et al. [9,32] and Castro et al. [16] reported that although a general increase in pressures was observed, the most evident changes took place in the midfoot and forefoot areas, or affected the toes. These results differed from our own, possibly due to the difference in the age of the participants. In the latter study, the participants were school-age children, whose postural control system might not yet be fully mature, meaning that the imposition of an external load could provoke a forward displacement of the centre of pressure. Another possible explanation concerns the anatomical zones of the foot taken into account in each study. In the present study, the pressures considered were only those exerted on the hindfoot, the forefoot and the full foot, whereas these earlier investigations also analysed the effects produced on the midfoot and the region of the toes. These factors might account for the differences in the results obtained. Furthermore, in calculating the backpack load, Castro et al. [16] did not use the percentage of body weight, but took a BMI of 30 kg/m^2^ as the maximum weight criterion.

An important finding of our study was that the pattern of loads differed between the feet; thus, a greater plantar pressure was exerted on the right foot than on the left, in the static analysis. In the dynamic test, however, no such differences were observed. These results contrast with those obtained by Balkó et al. [26], who reported distinct load patterns between dominant and non-dominant limbs during propulsion and braking. In our sample, the Foot Posture Index (FPI) indicated an average neutral to mildly pronated alignment, with no dominance-related asymmetries. This suggests that the inter-limb differences observed in static testing may reflect individual postural strategies rather than true functional dominance.

Our study results indicate that sex is not a relevant factor in the discrepancy of plantar pressure loads. This contrasts with Chen et al. [33] who observed differences in this respect between male and female subjects, although in the latter case the sample size (*n* = 20) was insufficient to assume statistical significance. Let us recall that the main aim of the present study was to determine safe, appropriate weights for load carrying by hikers and to assess the alterations produced in plantar pressure by variations in this load.

The main contribution of this study is the identification of a preliminary, sample-specific reference point for load-induced plantar pressure increase. According to the *t*-test results, a static pressure increase of approximately 5.33% corresponds to carrying a load equivalent to 10% of body weight. Dynamic analysis confirmed that exceeding this load leads to significant increases in plantar pressure across all foot regions. These findings suggest that loads above ~10% body weight may coincide with a shift toward higher plantar loading demands under the present testing conditions. Therefore, this value should be interpreted as an exploratory benchmark rather than a universal “safe limit”, and longitudinal studies are needed to confirm whether this reference point predicts injury risk over time.

From a scientific standpoint, these findings help define quantitative limits of plantar adaptation to external load. Identifying the point at which compensatory mechanisms are potentially challenged a biomechanical basis for understanding overuse injuries in weight-bearing activities such as hiking, mountaineering, or military marching [5,15] This work therefore addresses a general problem of functional load tolerance rather than footwear-specific performance.

Although participants were assessed under controlled conditions, cumulative fatigue from long-distance walking may have influenced plantar loading and gait patterns. While this reflects real hiking demands and enhances ecological validity, future studies including repeated assessments or field-based protocols could clarify the extent to which fatigue modulates load-induced biomechanical responses.

### Limitations

This study is subject to some limitations. Firstly, the parameters considered were measured with the hikers standing or walking barefoot, a situation that obviously does not represent the actual conditions experienced while hiking. Future studies should use instrumented insoles to measure plantar pressures with footwear. Although foot posture (FPI-6) was assessed, specific foot deformities and detailed clinical diagnoses were not recorded. The simultaneous increase in contact area and plantar.

A non-hiker control group was not included; therefore, we cannot determine whether the observed load-related plantar pressure responses are specific to long-distance hikers or reflect general adult responses to load carriage. The findings should be interpreted as within-subject responses in recreational adults under ecologically realistic long-distance walking conditions.

Another potential source of bias is walking speed, which was self-selected and not recorded. However, this approach is widely used in gait research to ensure ecological validity and prevent alterations in natural gait patterns. Nevertheless, this decision may have introduced some interindividual variability.

Additionally, testing followed a fixed order, which could have introduced potential fatigue or learning effects, although rest periods were provided between trials to minimize this risk.

The relatively small sample size (*n* = 39) also limits the generalizability of the results, as participants were active adults who had recently completed a long-distance walk. Future studies should include larger and more homogeneous or stratified samples and consider additional variables such as foot type, dominance, or ankle range of motion, which may influence plantar load distribution.

In addition, the heterogeneous age range may influence plantar pressure and spatiotemporal gait parameters; therefore, the present findings should be interpreted as within-subject responses under the tested conditions rather than population-level estimates [22]. Future work with larger samples could incorporate covariates/stratification (e.g., age groups, foot type) to improve external validity and enable more robust inference.

Despite these limitations, this research opens new avenues for investigation and provide a basis for future work.

Overall, this cross-sectional study provides preliminary evidence that loads exceeding approximately 10% of body weight are associated with higher plantar pressures under the present protocol. Longitudinal and field-based analyses are warranted to validate this threshold and explore its relationship with overuse injury risk during long-distance walking.

## 5. Conclusions

The present study examined how external load influences plantar pressure distribution during walking. The results showed a progressive, load-dependent increase in plantar forces, with greater pressure consistently recorded in the hindfoot compared to the forefoot and slightly higher values in the right foot. An exploratory reference point around 10% of body weight was observed, beyond which plantar pressures increased significantly (3.3% in the forefoot, 5.2% in the hindfoot, and 4.6% for the full foot). These results provide baseline biomechanical data that may serve as a reference for future research on load tolerance during walking. Future studies with larger and stratified samples are warranted to validate this reference point across different age groups and hiking contexts.

## Figures and Tables

**Table 1:** Sociodemographic and anthropometric characteristics for the total sample, by sex.

Variable	Men (*n* = 21) Mean ± SD (Range)	Women (*n* = 18) Mean ± SD (Range)	Total (*n* = 39) Mean ± SD (Range)	*p*-Value
Age (years)	39.80 ± 15.01 (21, 72)	32.83 ± 11.95 (22, 60)	36.57 ± 13.96 (21, 72)	0.148
Weight (kg)	79.58 ± 13.07 (65, 111)	60.79 ± 14.05 (47, 95)	70.68 ± 13.40 (47, 111)	<0.001 *
Height (cm)	177.14 ± 7.00 (167, 192)	163.00 ± 6.50 (152, 177)	170.61 ± 9.79 (152, 192)	<0.001 *
BMI	23.03 ± 6.77 (18.67, 32.93)	24.20 ± 4.39 (18.67, 33.63)	23.57 ± 5.75 (18.10, 33.63)	0.022 *
FPI (left foot)	0.67 ± 3.45 (−6, 6)	2.89 ± 3.34 (−4, 8)	1.69 ± 3.54 (−6, 8)	0.049 *
FPI (right foot)	1.19 ± 3.43 (−6, 6)	2.89 ± 3.07 (−2, 8)	1.97 ± 3.34 (−6, 8)	0.114

BMI, body mass index; SD, standard deviation; FPI, Foot Posture Index. Height and Weight followed normal distributions (Kolmogorov–Smirnov test), and Student’s *t*-test was used to assess sex-related differences. Age and BMI did not follow normal distributions; therefore, the Mann–Whitney U test was applied. In all analyses, * *p* < 0.05 (95% confidence interval) was considered statistically significant. The Foot Posture Index (FPI) was recorded to characterize the podiatric profile of the participants. Positive values indicate pronated foot posture, negative values indicate supinated posture, and values near zero correspond to neutral alignment.

**Table 2:** Excessive or appropriate backpack load carried, with respect to the recommended limit of 10% of body weight.

	Men (*n* = 21) *n* (%)	Women (*n* = 18) *n* (%)	Total (*n* = 39) *n* (%)
Equal to the 10% recommended limit	1 (4.8)	1 (5.6)	2 (5.1)
Backpack weight: >10% of body weight (Excessive)			
0.1–1.0 kg above limit	5 (23.8)	3 (16.7)	8 (20.5)
1.1–2.0 kg above limit	1 (4.8)	1 (5.6)	2 (5.1)
2.1–3.0 kg above limit	5 (23.8)	3 (16.7)	8 (20.5)
3.1–4.0 kg above limit	0 (0)	3 (16.7)	3 (7.7)
≥4.1 kg above limit	1 (4.8)	4 (22.2)	5 (12.8)
Backpack weight: <10% of body weight (Correct)			
0.1–1.0 kg below limit	3 (14.3)	1 (5.6)	4 (10.3)
1.1–2.0 kg below limit	3 (14.3)	2 (11.1)	5 (12.8)
2.1–3.0 kg below limit	1 (4.8)	0 (0)	1 (2.6)
3.1–4.0 kg below limit	1 (4.8)	0 (0)	1 (2.6)

**Table 3:** Pressure increases in the forefoot, hindfoot, and whole foot in a static position with a backpack weighing 10% and 20% of body weight compared to no backpack.

Variables	% Increase in Pressure Carrying a Backpack with 10% of Body Weight Compared to No Backpack	% Increase in Pressure Carrying a Backpack with 20% of Body Weight Compared to No Backpack	*p*-Value
Forefoot	5.41 ± 6.08	8.73 ± 3.65	<0.001
Hindfoot	5.01 ± 8.01	10.17 ± 10.17	0.001
Total foot	5.04 ± 6.38	9.61 ± 6.31	<0.001

Data are expressed as mean ± standard deviation (SD) for 39 participants. The *p*-value indicates the statistical difference in pressure increase between the 20% and 10% backpack conditions. Results reflect the relative rise in plantar loading across foot regions with incremental backpack weight.

**Table 4:** Paired *t*-test results and effect sizes comparing each backpack condition with the no-backpack reference.

Variable	Condition	*p*-Value	Cohen’s d (Effect Size)
Maximum pressure (g/cm^2^)	Habitual	0.312	0.16 (Small)
	10% body weight	0.013 *	0.42 (Medium)
	20% body weight	0.032 *	0.36 (Small–Medium)
Mean pressure (g/cm^2^)	Habitual	<0.001 *	0.83 (Large)
	10% body weight	0.002 *	0.54 (Medium)
	20% body weight	0.038 *	0.34 (Small–Medium)

*p*-values and effect sizes (Cohen’s d) obtained from paired Student’s *t*-tests comparing each loaded condition (habitual, 10%, and 20% of body weight) with the no-backpack reference. Effect sizes were calculated numerically using the formula d = t/√n for paired samples (*n* = 39). Conventional thresholds (0.2 = small, 0.5 = medium, 0.8 = large) were used to interpret magnitude. * *p* < 0.05.

**Table 5:** Descriptive statistics of plantar pressure and contact surface area in the unloaded condition and corresponding percentage variation tolerated without significant difference.

Variable	No Backpack	Maximum Non-Significant Difference (%)
Maximum pressure (g/cm^2^)	1736 ± 285.40 (1244, 2477)	1828.52 (5.33%)
Mean pressure (g/cm^2^)	Left foot: 1075.51 ± 432.76 Right foot: 1064.10 ± 450.97	Left foot: 1215.80 (13.04%) Right foot: 1210.29 (13.74%)
Surface area (cm^2^)	Left foot: 135.87 ± 22.57 Right foot: 138.33 ± 24.43	Left foot: 143.19 (3.15%) Right foot: 145.93 (5.49%)

Data are expressed as mean ± standard deviation (SD) and range. The column “Maximum non-significant difference (%)” represents the upper boundary of variation tolerated without statistical significance, as determined through a one-sample Student’s *t*-test using the unloaded condition as reference. These values define the biomechanical tolerance threshold for plantar pressure and surface area changes under incremental loading.

## Data Availability

The data are available from the corresponding author upon reasonable request. The data are not publicly available due to restrictions (privacy/ethical and institutional agreements).

## Supplementary Materials

The following supporting information can be downloaded at: https://www.mdpi.com/article/10.3390/jfmk11010036/s1.

## Abbreviations

The following abbreviations are used in this manuscript:

BMI	Body Mass Index
SD	Standard Deviation
SPSS	Statistical Package for the Social Sciences
STROBE	Strengthening the Reporting of Observational Studies in Epidemiology
