# Supplementary material for: Plantar Pressure Responses to Backpack Load in Long-Distance Hikers: A Cross-Sectional Observational Study
Source: J Funct Morphol Kinesiol. 2026 Jan 15;11(1):36. doi: 10.3390/jfmk11010036 (PMC12821408; doi:10.3390/jfmk11010036)
Supplement: Supplementary file 1 [file jfmk-11-00036-s001.zip › jfmk-4015411-supplementary.pdf]

Supplementary Materials

Supplementary Material 1

Table S1. Descriptive statistics of plantar surface area, vertical force, load distribution, and pressure exerted on the forefoot, hindfoot, and total foot during static measurements under different backpack load conditions.

		Right foot N=39				Left foot N=39			
		No backpack Mean±SD (Range)	Habitual backpack Mean±SD (Range)	Backpack with 10% body weight Mean±SD (Range)	Backpack with 20% body weight Mean±SD (Range)	No backpack Mean±SD (Range)	Habitual backpack Mean±SD (Range)	Backpack with 10% body weight Mean±SD (Range)	Backpack with 20% body weight Mean±SD (Range)
Forefoot	Surface area (cm²)	69.71±13.65 (43.33-108.66)	72.01±14.14 (40.66-116.33)	73.87±14.18 (40.66-111.66)	77.53±14.85 (51.33-123)	69.65±13.26 (40.33-105)	72.20±13.04 (40.66-112)	73.11±12.79 (40.66-107.33)	77.04±14.93 (49.66-122.66)
	Force (%)	24.17±4 (16-33)	28.93±32.69 (17.33-226.66)	24.10±3.60 (16.33-31)	24.25±3.14 (18.33-30)	23.24±3.64 (16-31.66)	23.57±3.43 (17.33-31.33)	26.64±3.53 (15.66-34)	23.78±2.97 (15-31)
	Distribution (%)	47.77±7.36 (36-66.66)	47.45±6.63 (34.33-65.66)	48.09±6.67 (34.33-66.33)	48.41±6.01 (36.33-64.33)	46.82±6.40 (34.33-58.66)	47.16±6.30 (35-61)	47.44±6.92 (32.66-64.33)	47.72±5.78 (31.33-59.33)
	Pressure (kg/cm²)	0.243±0.035 (0.18-0.31)	0.258±0.034 (0.19-0.33)	0.251±0.036 (0.19-0.34)	0.264±0.039 (0.20-0.36)	0.234±0.033 (0.18-0.32)	0.256±0.038 (0.18-0.35)	0.249±0.035 (0.18-0.37)	0.260±0.036 (0.17-0.37)
Hindfoot	Surface area (cm²)	66.53±14.27 (39.33-93.66)	68.23±13.78 (39.66-98)	69.38±14.32 (40.66-101.66)	71.96±15.74 (43-107)	64.24±14.49 (39.33-95)	66.86±13.10 (43-99.66)	66.83±13.18 (43-92.33)	64.40±14.27 (44.33-102.33)
	Force (%)	27.10±5.87 (15.33-54.33)	27.02±5.95 (16.66-56.66)	26.88±5.86 (15.66-56)	57.66±26.57 (16.66-57.66)	26.24±3.17 (20.33-33.33)	26.33±3.2 (19.33-32.26)	26.16±3.60 (19.33-33)	26.13±3.12 (19.66-32.66)
	Distribution (%)	54.31±15.31 (33.33-136)	54.44±14.12 (34.33-130.33)	53.46±13.76 (33.66-126.66)	53.68±15.11 (35.66-138.33)	53.16±6.40 (41.33-65.66)	52.81±6.30 (39-65)	52.59±6.73 (35.66-67.33)	52.26±5.80 (40.33-68.66)
	Pressure (kg/cm²)	0.293±0.101 (0.16-0.82)	0.317±0.108 (0.20-0.88)	0.307±0.111 (0.20-0.90)	0.318±0.105 (0.21-0.88)	0.291±0.052 (0.20-0.42)	0.312±0.55 (0.19-0.46)	0.305±0.59 (0.17-0.48)	0.320±0.55 (0.21-0.43)
Full foot	Surface area (cm²)	135.50±25.17 (82.66-202.33)	140.46±26.19 (84.33-213.66)	143.24±27.01 (84.33-213.33)	149.52±29.02 (102.33-219.66)	133.91±25.74 (79.66-197)	139.22±24.83 (83.66-211.66)	140.02±23.94 (83.66-197.33)	146.50±27.80 (102-225)
	Force (%)	50.58±2.83 (43.66-58.66)	50.05±2.31 (46.33-55.33)	50.20±2.51 (44.33-54.66)	50.08±2.01 (46.33-53.66)	49.45±2.88 (41.33-56.33)	49.97±2.32 (44.66-53.66)	49.79±2.50 (45.33-55.66)	49.92±2 (46.33-53.66)
	Distribution (%)	35.67±7.99 (23-56)	39.33±8.66 (25.66-63.33)	39.10±9.02 (25-62.66)	42.45±10 (27.33-69.66)	35.16±8.5 (23-57.66)	39.27±8.93 (26-59.66)	38.63±8.75 (24.66-62)	42.28±9.88 (27-65.33)
	Pressure (kg/cm²)	0.261±0.034 (0.21-0.33)	0.280±0.39 (0.21-0.38)	0.273±0.039 (0.20-0.37)	0.284±0.040 (0.21-0.39)	0.262±0.35 (0.21-0.34)	0.282±0.40 (0.21-0.41)	0.275±0.4 (0.20-0.42)	0.288±0.39 (0.22-0.40)

Data are presented as mean±standard deviation (SD) and range for both feet (n = 39). The variables include surface area (cm²), percentage of vertical force distribution (%), and pressure (kg/cm²) in the forefoot, hindfoot,

and full-foot regions. Conditions correspond to unloaded (no backpack), habitual backpack, backpack with 10% of body weight, and backpack with 20% of body weight.

Supplementary Material 2. **Table S2.** Descriptive statistics of maximum and mean plantar pressure and contact surface area for the right and left foot under different backpack load conditions.

	Right foot (N=39)					Left foot (N=39)				
	No backpack	Habitual backpack	Backpack with 10% body weight	Backpack with 20% body weight	Maximum non-significant difference (%)	No backpack	Habitual backpack	Backpack with 10% body weight	Backpack with 20% body weight	Maximum non-significant difference (%)
Maximum pressure (g/cm²)	1736±285.40 (1244,2477)	1788.89±290.55 (1174,2510)	1791.76±379.83 (1133,2955)	1861.68±428.39 (259,2976)	1828.52 **(5.33%)**	1736±285.40 (1244,2477)	1824.84±404.18 (1174,3578)	1772.29±340.76 (1133,2631)	1854.08±429 (259,2976)	1828.52 **(5.33%)**
Mean pressure (g/cm²)	1064.1±450.97 (601,3574)	1074.38±232.07 (761,1796)	1056.52±182.05 (760,1415)	1107.86±352.91 (126,2399)	1210.29 (13.74%)	1075.51±432.76 (691,3474)	1079.79±236.52 (741,1854)	1033.21±162.44 (774,1436)	1045.79±309.06 (82,1912)	1215.80 (13.04%)
Surface area (cm²)	138.33±24.43 (99,186)	144.76±23.03 (99,193)	143.29±21.04 (108,183)	146.18±22.21 (94,191)	145.93 (5.49%)	135.87±22.57 (88,185)	138.82±21.48 (93,184)	141.76±21.46 (109,188)	144.29±22.33 (112,195)	143.19 (3.15%)

Data are expressed as mean±standard deviation (SD) and range. The column "Maximum non-significant difference (%)" indicates the upper limit of variation tolerated without reaching statistical significance according to the one-sample Student's t-test, representing the biomechanical threshold of non-significant change
